# Phytochemical analysis and evaluation of its antioxidant, antimicrobial, and cytotoxic activities for different extracts of *Casuarina equisetifolia*

**DOI:** 10.1186/s12906-024-04422-4

**Published:** 2024-03-20

**Authors:** Walid Elsayed Abdallah, Khaled Ahmed Shams, Ashraf Moursi El-Shamy

**Affiliations:** 1 Chemistry of Medicinal Plants Department, Pharmaceutical and Drug Industries Research Institute, National Research Centre, 33 El Buhouth St. (FormerEl Tahrir St.), Dokki, Giza, 12622 Egypt; 2 Physical Chemistry Department, Electrochemistry and Corrosion Lab, National Research Centre, El-Bohouth St. 33, Dokki, Giza, 12622 Egypt

**Keywords:** *Casuarinaceae*, *C. equisetfolia*, Phenolics, Antioxidant, Antimicrobial, Cytotoxic activity

## Abstract

**Background:**

*Casuarina equisetifolia* belongs to the *Casuarina* species with the most extensive natural distribution, which contain various phytochemicals with potential health benefits. This study aimed to investigate the chemical composition and biological activities of different extracts of *Casuarina equisetifolia.*

**Methods:**

The *n*-hexane extract was analyzed for its unsaponifiable and fatty acid methyl esters fractions, while chloroform, ethyl acetate, and butanol extracts were studied for their phenolic components. Six different extracts of *C. equisetifolia* needles were evaluated for their total phenolic content, total flavonoid content, and their antioxidant, antimicrobial, and cytotoxic activities.

**Results:**

The *n*-hexane extract contained mainly hydrocarbons and fatty acid methyl esters, while ten phenolic compounds were isolated and identified in the chloroform, ethyl acetate, and butanol extracts. The methanolic extract exhibited the highest total phenolic and flavonoid content, highest antioxidant activity, and most potent cytotoxic activity against HepG-2 and HCT-116 cancer cell lines. The ethyl acetate extract showed the most significant inhibition zone against *Staphylococcus aureus* and *Bacillus subtilis*.

**Conclusion:**

*Casuarina equisetifolia* extracts showed promising antioxidant, antimicrobial, and cytotoxic activities. Overall, *Casuarina equisetifolia* is a versatile tree with a variety of uses, and its plant material can be used for many different purposes.

## Background

Cancer is a complex disease that affects public health globally. Naturally occurring compounds found in vegetables and herbs, such as alkaloids, terpenoids, and polyphenols, have antitumor and chemopreventive effects. Up to 70% of approved anticancer agents have been derived from natural sources [1]. *Casuarina equisetifolia* is an evergreen plant belonging to the *Casuarinaceae* family that grows in various environmental conditions and is known to store various compounds, including catechin, ellagic acid, gallic acid, quercetin, and lupeol [2]. The presence of diverse compounds, including carbohydrates, alkaloids, proteins, glycosides, saponins, phenolics, flavonoids, tannins, steroids, gum, reducing sugars, and triterpenoids, was displayed by phytochemical screening of several *Casuarina equisetifolia* parts [3]. Several compounds have been isolated from *Casuarina equisetifolia*, including kaempferol-3-α-rhmanoside, quercetin-3-α-araboside, luteolin-3',4'-dimethoxy-7-*β*-rhamnoside, kaempferol-3-*β*-dirhamnoside, and quercetin-3-*β*-glucoside [3]. Additionally, compounds such as 6,7-dimethoxy coumarin and scopoletin have been isolated from the chloroform-soluble fraction of methanol extract of the plant's fresh leaves [4]. HPLC analysis has shown that the plant contains phenolic and flavonoidal compounds, including gallic acid, protochatechuic acid, quercetin, rutin, and kampferol [5]. *Casuarina equisetifolia* has a long history of traditional medicinal use, with various parts of the plant being used to treat a range of ailments including respiratory, digestive, and skin disorders [6]. Recent scientific research has focused on the plant's phytochemical profile and the assessment of its antioxidant, antimicrobial, and cytotoxic activities [7–9]. Flavonoids are a group of polyphenolic compounds that are widely distributed in the plant kingdom and are known for their antioxidant activity. Investigation of the antioxidant activity of different extracts of *Casuarina equisetifolia* using various *in vitro* assays, including the DPPH assay, the FRAP assay, and the ABTS assay [10]. Several studies have investigated the antimicrobial activity of the plant extracts against a range of bacteria and fungi, including *Staphylococcus aureus*, *Escherichia coli*, *Candida albicans*, and *Aspergillus niger* [11]. *Casuarina equisetifolia* extracts have significant cytotoxic activity against different cancer cell lines, like breast cancer, lung cancer, and colon cancer cell line [12, 13]. The phytochemical profile and bioactivity of different parts of the plant, including the leaves, bark, and wood have been investigated [14]. According to our understanding, limited work has been carried out on the antiproliferative activity of *Casuarina equisetifolia* needles, this study aims to isolate some chemical components and investigate the *in vitro* antioxidant, antimicrobial, and antiproliferative activities of different extracts of the plant's needles.

## Materials and methods

### Plant material

In March 2022, needles from the *C. equistefolia* plant were collected from an authorized farm in Zagazig city, El-Sharkia governorate in Egypt. The Collection of the plant was done according to the National Research Centre in Egypt following national institutional guidelines and regulations. The identification of the plant was done by Dr. Mona Marzouk, who is a Professor of Taxonomy at the National Research Centre in Egypt. A voucher specimen of the plant was kept in the herbarium of the National Research Centre with accession number M194. The above-ground part of the plant was dried in the shade for 10 days, with a daily reversal. Once completely dried, the plants were finely powdered to facilitate extraction and analysis.

## Extraction and fractionation of *C. equistefolia* needles

A total of 1.5 kg of air-dried powdered *C. equistefolia* needles were extracted exhaustively using 9 L of 80% methanol through percolation. The resulting methanol extract was then concentrated under reduced pressure, resulting in a residue of 160 g. This residue was dissolved in 750 mL of hot distilled water and left to stand overnight. The filtrate was then subjected to sequential partitioning with hexane (3 x 500 mL), chloroform (3 x 500 mL), ethyl acetate, n-butanol (3 x 500 mL), and the residue of the mother liquor fraction.

### Isolation of lipoidal matter

A portion of the hexane extract that was obtained was subjected to saponification, which resulted in the production of unsaponifiable matter and fatty acid methyl esters.

About 2 g of the hexane extract were saponified by refluxing with 100 mL N/2 alcoholic KOH. The unsaponifilable matters were extracted by shaking with successive portions of diethylether (3×100 ml). The combined ether extract was evaporated *in vacuo* till dryness to give a yellowish semi solid residue of unsaponifiable matters which were subjected to GC/MS analysis. The hydroalcoholic soap solution after saponification was rendered acidic (pH-2) with 5% sulphuric acid (H_2_SO_4_). The liberated fatty acids were thoroughly extracted several times with diethylether. The solvent was evaporated *in vacuo* at about 40 °C till dryness. GC/MS analysis of the fatty acid methyl esters was carried out [15].

### GC/MS analysis of unsaponifiable fraction

GC/MS analysis of unsaponifiable fraction refers to the use of gas chromatography (GC) coupled with mass spectrometry (MS) to analyze the non-saponifiable components of a sample, such as oils or fats.

Gas chromatography-mass spectrometry was used to analyze the unsaponifiable fraction of *C. equisetifolia* needles at the Department of Medicinal and Aromatic Plants Research, National Research Center. The equipment used for the analysis included a TRACE GC Ultra Gas Chromatographs (THERMO Scientific Corp., USA), coupled with a thermo mass spectrometer detector (ISQ Single Quadrupole Mass Spectrometer) and a TG-5MS column (30 m x 0.25 mm i.d., 0.25 μm film thickness). Helium was used as the carrier gas at a flow rate of 1.0 mL/min and a split ratio of 1:10. The temperature program used was 50°C for 3 min, followed by a rise of 5.0°C /min to 300°C and holding for 20 min. The injector and detector were maintained at 280°C.

### GC/MS analysis for FAMEs

The analysis of the fatty acid methyl esters (FAMEs) of *C. equisetifolia* needles was conducted using a temperature program that began at 80°C for 1 minute, followed by a rise of 4.0°C /min to 300°C and held for 1 minute. The injector and detector were set to 240°C, and the diluted samples (1:10 hexane, v/v) of 0.2 μL of the mixtures were injected. Helium was used as the carrier gas at a flow rate of 1.0 mL/min and a split ratio of 1:10. Mass spectra were obtained by electron ionization (EI) at 70 eV, using a spectral range of m/z 40-450. The National Institute of Standards and Technology (NIST) reference library, Willey 5, and mass finder were used to interpret the mass spectra, along with data reported by Adams. The constituent percentages were determined based on the peak area.

### Fractionation and isolation of the phenolic components of *C. equistefolia* extracts

The chloroform extract weighing 1.3 g was passed through a silica gel column chromatography with 230-400 mesh size, using an n-hexane-EtOAc mixture with increasing polarity to obtain 31 fractions of 100 mL each. The progress of the fractionation process was monitored using paper chromatography on Whatman 1 mm with n-butanol: acetic acid: water (BAW) (3:1:1) and 15% acetic acid as solvent systems. Fractions 2-7 were combined and subjected to further separation on a silica gel column chromatography with n-hexane/EtOAc (10:1, v/v) to isolate compound 1 (43 mg). Similarly, fractions 10-12 and 16-24 were pooled together and subjected to column chromatography using n-hexane/EtOAc (v/v) in ratios of 10:1 and 7:3, respectively, to yield compounds 2 and 3, weighing 29 mg and 43 mg, respectively. For the ethyl acetate fraction weighing 2.1 g, a Sephadex LH-20 column (100 x 5 cm) was used with methanol: water (90:10) as the solvent system to obtain 38 fractions of 100 mL each. These fractions were then combined based on their similarity, resulting in four main sub-fractions, consisting of sub-fractions 4-9, 13-16, 18-24, and 27-33, respectively. The sub-fraction 4-9 (0.22 g) was subjected to silica gel column chromatography (50 x 3 cm) using chloroform and chloroform-methanol gradients. The main fraction obtained was further purified on a Sephadex LH-20 column (50 x 3 cm) to isolate compound 4 (25 mg). Similarly, the 13-16 sub-fraction (0.3 g) was purified using silica gel column chromatography (50 x 3 cm) with chloroform: methanol (95:5) as the eluent to yield compound 5 (30 mg). The 18-24, and 27-33 sub-fractions (0.2 g) were subjected to preparative paper chromatography using n-butanol: acetic acid: water (3:1:1) as the solvent system. The two main bands were isolated, and the compounds were further purified using a Sephadex LH-20 column eluted with methanol: water (95:5) to obtain compounds C-6 (20 mg) and C-7 (35 mg). The butanol fraction weighing 3.1 g was passed through a Polyamide column (100 x 5 cm) using water, water/methanol gradients, and methanol 100% to obtain fractions of 250 mL each. These fractions were then subjected to paper chromatographic investigation on Whatman 1 mm using BAW (3:1:1) and 15% acetic acid as solvent systems. The fractions exhibiting similar chromatographic patterns were pooled together, and the main compounds were further purified using small columns of Polyamide using water/methanol gradients and methanol 100%, and Sephadex LH-20 eluted with methanol: water (95:5) to yield three compounds (C-8, C-9, and C-10). The purity of the isolated compounds was verified using TLC and PC.

### Phytochemical screening

Quantitative analyses were conducted to examine the phytochemicals present in six distinct extracts from *C. equistefolia*, namely chloroform extract (7 CE), ethyl acetate extract (8 CE), n-butanol extract (9 CE), methanol extract (10 CE), aqueous mother liquor (11 CE), and hexane extract (12 CE). Studies have shown that *Casuarina equisetifolia* contains several classes of phytochemicals such as flavonoids, tannins, alkaloids, terpenoids, and phenolic compounds. These phytochemicals are known to exhibit various biological activities such as antioxidant, antimicrobial, and cytotoxic properties. Overall, the phytochemical screening of different extracts of *Casuarina equisetifolia* can help to identify the presence of various bioactive compounds that may have potential pharmacological activities.

### Total phenolic contents (TPC)

Using gallic acid as a reference standard, the Folin-Ciocalteu method was employed to evaluate the total phenolic contents of the six distinct extracts [16].

### Total flavonoid contents (TFC)

The total flavonoid contents of the six different extracts was assessed using the colorimetric method described by [17], with quercetin as the reference standard. The TFC was expressed in terms of quercetin equivalents per gram of extract.

### Biological studies

#### Antioxidant activity assay

The DPPH method, as described by [18], was employed to determine the radical scavenging activity of the six different plant extracts. The assay mixture consisted of 1 mL of 0.1 mM DPPH (dissolved in methanol), and 1 mL of extract (dissolved in 10 to 500 µg/mL methanol), and the volume was made up to 3 mL with methanol. The mixture was thoroughly shaken and left in the dark at normal room temperature for 30 minutes. The absorbance was measured at 517 nm using a blank solution (1 mL sample + 2 mL methanol). Quercetin was used as the standard compound. The antioxidant activity of the plant extracts was calculated using the following formula:

1 $$\mathrm{Antioxidant \,activity }(\mathrm{\%}) = 100 - \{[(\mathrm{AB\, sample }-\mathrm{ AB\, blank})\mathrm{ x }100] /\mathrm{ AB\, control}\}$$

Where AA represents antioxidant activity, AB sample represents the absorbance of the sample and DPPH, AB blank represents the absorbance of the sample and methanol, and AB control represents the absorbance of DPPH and methanol.

#### Antimicrobial activity

The antimicrobial activity of six different extracts was evaluated against various microorganisms using a modified Kirby-Bauer disc diffusion method, following the protocol described by [19]. Six microorganisms, including bacterial and fungal strains obtained from the Micro Analytical Center at the Faculty of Science, Cairo University, were investigated; *Staphylococcus aureus* (ATCC 12600) and *Bacillus subtilis* (ATCC 6051) were used as Gram-positive bacteria, while *Escherichia coli* (ATCC 11775) and *Pseudomonas aeruginosa* (ATCC 10145) were used as Gram-negative bacteria. *Candida albicans* (ATCC 7102) and *Aspergillus flavus* (ATCC 9643) were used as fungal strains. Ampicillin and Amphotericin B (Bristol-Myers Squibb, Switzerland) were used as standard antibacterial and antifungal drugs, respectively. Filter discs impregnated with 10 µL of solvent (distilled water, chloroform, DMSO) were used as negative controls. The zone of inhibition, or clear zone, was designated as the region with no growth around the disc. 100 µl of microbial suspension was spread onto agar plates. The approved standard disc diffusion technique for filamentous fungi (NCCLS, 2002) was used to investigate the susceptibilities of filamentous fungi to antifungal agents. The approved standard method for yeasts (NCCLS, 2003) was used. Plates inoculated with *A. flavus* were incubated at 25°C for 48 hours. *S. aureus, B. subtilis, E. coli*, and *P. aeruginosa* were incubated at 35-37°C for 24-48 hours. *C. albicans* was incubated at 30°C for 24-48 hours, and the diameters of the inhibition zones were measured [20].

#### Cytotoxic activity

At Al-Azhar University, The Regional Center for Mycology & Biotechnology, the assessment of the impact on cell viability for six distinct extracts was conducted using HepG-2 and HCT-116 cell lines. The HepG-2 cells, which are a type of human hepatocellular cancer cell line, and the HCT-116 cells, which are colon carcinoma cells, were procured from VACSERA Tissue Culture Unit.

#### Chemicals used

Sigma (St. Louis, Mo., USA) was the supplier of Dimethyl sulfoxide (DMSO), crystal violet, and trypan blue dye. Lonza was the source of Fetal Bovine serum, DMEM, RPMI-1640, HEPES buffer solution, L-glutamine, gentamycin, and 0.25% Trypsin-EDTA. Crystal violet stain (1%) was prepared by combining 0.5% (w/v) crystal violet and 50% methanol, followed by adjusting the final volume with ddH_2_O and filtering it through a Whatmann No.1 filter paper.

#### Cytotoxicity assay

To conduct the cytotoxicity assay, cells were seeded into a 96-well plate at a concentration of 1×104 cells per well, with 100 µL of growth medium added to each well. After 24 hours, a fresh medium containing various test samples and vinblastine sulfate as a positive control were added. The test samples were added as two-fold dilutions to the confluent cell monolayers dispensed into 96-well, flat-bottomed microtiter plates (Falcon, NJ, USA). The microtiter plates were then placed in a humidified incubator with 5% CO_2_ at 37ºC for 48 hours. Three wells were allocated for each test sample concentration, with control cells being incubated without test samples and with or without DMSO. It was observed that the small amount of DMSO present in the wells (maximum 0.1%) did not impact the experiment. To assess cell viability, a colorimetric method was used, as previously reported by [21]. Following aspiration of the media, 1% crystal violet solution was added to each well and left for 30 minutes, with the procedure completed following [22]. Cytotoxicity refers to the ability of a substance to kill or damage cells.

### Statistical analysis

The study's outcomes were presented as mean ± standard deviation (S.D). The data were analyzed and compared using one-way analysis of variance (ANOVA) using Graphpad Prism software (San Diego, CA. USA).

## Results

### Identification of chemical constituents in hexane extract

#### Unsaponifiable fraction

**Fig. 1:**
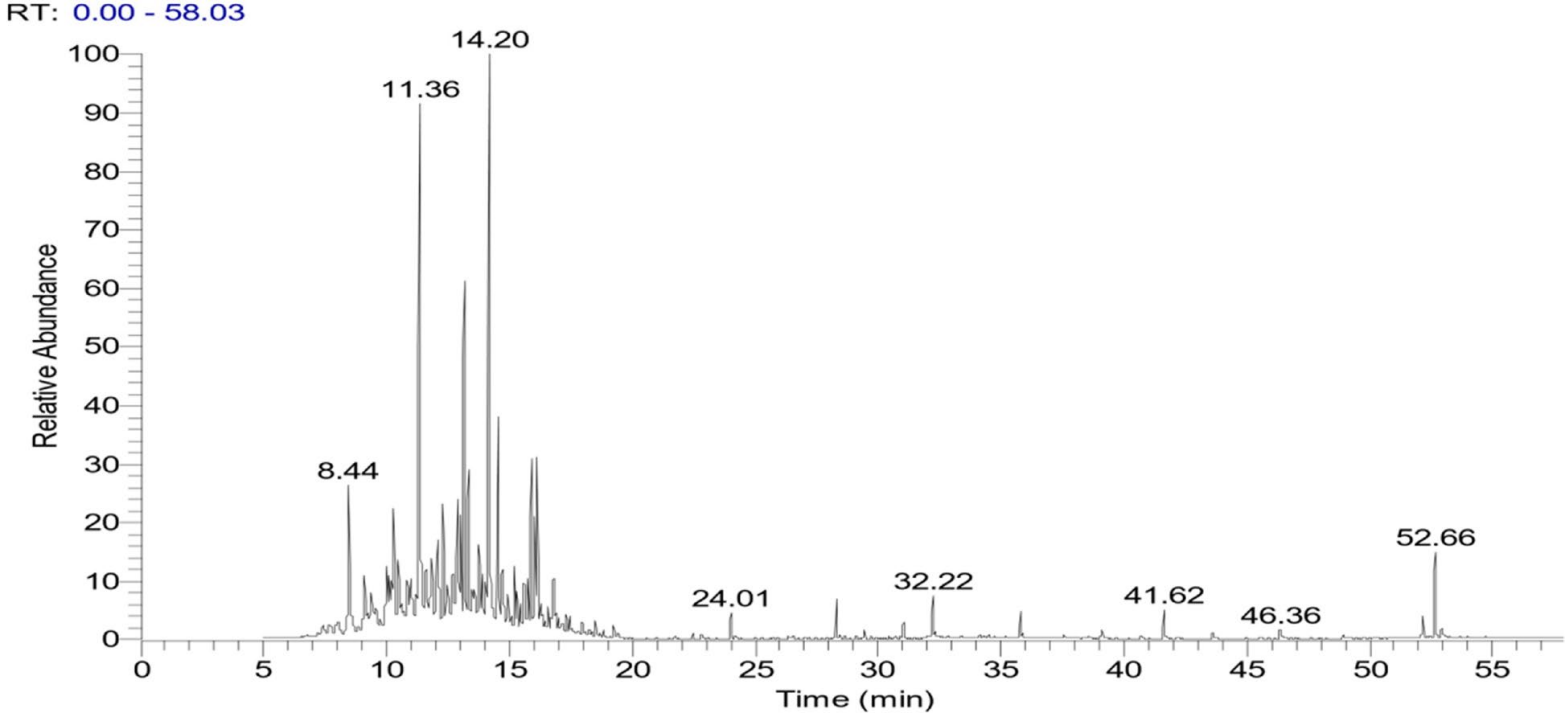
GC/MS chromatogram of the unsaponifiable fraction of *C. equistefolia* needles

The analysis of unsaponifiable fraction (UF) can provide valuable insights into the composition and potential health and industrial applications of various lipids and oils. Gas chromatography/mass spectrometry (GC/MS) was utilized to analyze the unsaponifiable fraction of *C. equistefolia* needles. The total ion chromatogram and the various components of the unsaponifiable fraction were shown in Fig. 1. The unsaponifiable fraction's GC/MS analysis (Table 1) revealed that it included a combination of triterpenes, sterols, and hydrocarbons. The hydrocarbons ranged from C_6_ to C_35_, with C_7_ (16.15%) being the predominant hydrocarbon. The sterol α-sitosterol was also present (0.53%). The triterpenes α-amyrin, germanicol, and lupenone were present, with lupeol being the primary triterpene (9.09%).

**Table 1:** GC/MS of the unsaponifiable fraction of *C. equistefolia* needles

**Peak No.**	**RT (min)**	**Rel. %**	**M. Wt.**	**MF**	**Compounds**
1	8.44	3.54	142	C_10_H_22_	Decane
2	9.09	1.30	142	C_10_H_22_	Octane, 3,3-dimethyl
3	9.34	0.84	129	C_6_H_11_NO_2_	Cyclohexane, nitro-
4	9.98	1.44	138	C_10_H_18_	Cyclopentane, pentylidene-
5	10.10	1.76	156	C_11_H_24_	Decane, 3-methyl
6	10.19	0.44	156	C_11_H_24_	Decane, 4-methyl-
7	10.28	2.48	156	C_11_H_24_	Decane, 2-methyl-
8	10.84	1.07	126	C_9_H_18_	Cyclohexane,1-ethyl-4-methyl-, trans-
9	11.00	1.32	127	C_7_H_13_NO	Cyclohexanone, 2-methyl-, oxime
10	11.35	13.78	112	C_8_H_16_	1-Hexene, 3,4-dimethyl-
11	11.59	1.32	152	C_10_H_16_O	6-Octen-1-yn-3-ol, 3,7-dimethyl-
12	11.82	1.88	156	C_11_H_24_	Undecane
13	12.10	2.59	170	C_12_H_26_	Decane, 3,7-dimethyl-
14	12.30	2.59	140	C_9_H_16_O	5-Heptenal, 2,6-dimethyl-
15	12.69	1.03	238	C_16_H_30_O	Hexadecadien-7,11 ol-1
16	12.90	3.39	190	C_13_H_18_O	1-Hexen-4-ol, 3-methyl-5-phenyl-
17	13.02	1.74	170	C_12_H_26_	Undecane, 2-methyl
18	13.15	6.98	170	C_12_H_26_	Undecane, 4-methyl
19	13.33	2.77	170	C_12_H_26_	Undecane, 3-methyl
20	13.76	1.73	184	C_10_H_16_O_3_	Limonene
21	13.88	1.06	168	C_12_H_24_	6-Dodecene, (Z)-
22	14.20	16.15	141	C_7_H_15_N_3_	Heptane, 4-azido-
23	1454	3.62	184	C_13_H_28_	Undecane, 2,6-dimethyl-
24	14.75	0.84	186	C_12_H_26_O	1-Octanol, 2-butyl
25	15.20	1.10	168	C_12_H_24_	Cyclohexane, hexyl
26	15.30	0.74	140	C_10_H_20_	Cyclopentane, (3-methylbutyl)-
27	15.59	0.97	184	C_13_H_28_	Dodecane, 6-methyl
28	15.64	1.57	184	C_13_H_28_	Dodecane, 5-methyl
29	15.76	1.74	184	C_13_H_28_	Dodecane, 4-methyl
30	15.89	2.88	184	C_13_H_28_	Dodecane, 2-methyl
31	16.13	2.43	156	C_11_H_24_	Octane, 2,3,7-trimethyl
32	16.25	0.40	168	C_12_H_24_	4-Undecene, 6-methyl
33	16.85	0.94	184	C_13_H_28_	Tridecane
34	24.01	0.44	224	C_16_H_32_	Cetene
35	28.31	0.67	252	C_18_H_36_	E-7-Octadecene
36	32.22	0.72	280	C_20_H_40_	3-Eicosene
37	35.79	0.47	280	C_20_H_40_	9-Eicosene
38	46.36	0.86	492	C_35_H_72_	Pentatriacontane
39	52.15	1.53	414	C_29_H_50_O	α-Sitosterol
40	52.36	0.86	426	C_30_H_50_O	α-Amyrin
41	52.45	1.90	426	C_30_H_50_O	Germanicol
42	52.66	1.90	424	C_30_H_48_O	Lupenone
43	52.96	0.69	426	C_30_H_50_O	Lupeol

#### Fatty acid methyl esters

**Fig. 2:**
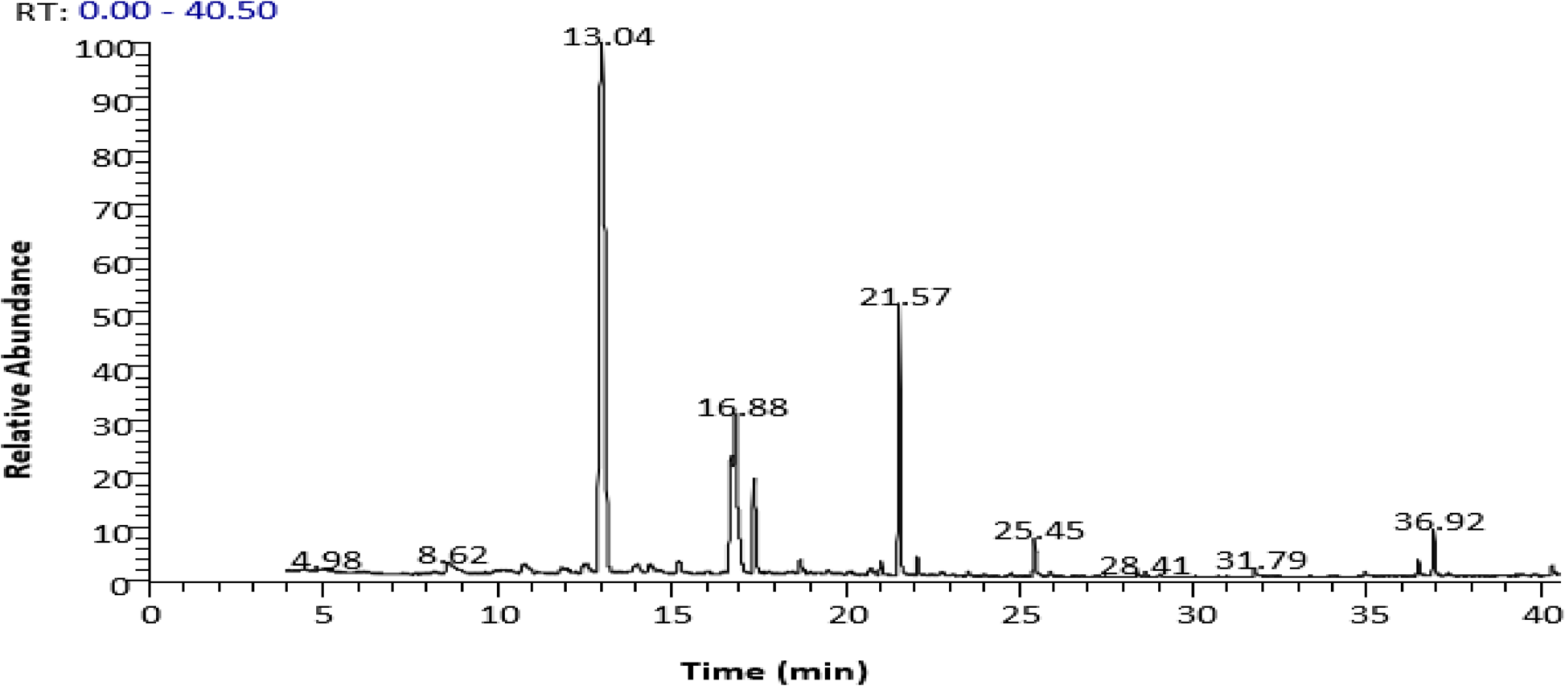
GC/MS Chromatogram of fatty acid methyl esters fraction of *C. equistefolia* needles

The GC/MS technique was used to examine the fatty acid methyl esters of *C. equistefolia*. The chromatogram (Fig. 2), depicted the analysis and presenting the fatty acid composition. The findings from the analysis of fatty acids using GC/MS (Table 2) showed that there were 13 fatty acid methyl esters present, which accounted for 89.44% of the overall acids. The primary fatty acid was palmitic acid methyl ester (C_16:0_), which constituted 54.75%, followed by arachidic acid methyl ester at 10.27%.

**Table 2:** GC/MS of fatty acid methyl esters fraction of *C. equistefolia* needles

**Peak No.**	**RT (min)**	**Rel. %**	**M. Wt.**	**MF**	**Compounds**
1	4.98	0.34	158	C_9_H_18_O_2_	Caprylic acid, methyl ester (C_8:0_)
2	8.62	0.82	242	C_15_H_30_O_2_	Myristic acid, methyl ester (C_14:0_)
3	13.04	54.75	270	C_17_H_34_O_2_	Palmitic acid, methyl ester (C_16:0_)
4	14.41	0.76	296	C_19_H_36_O_2_	Oleic acid, methyl ester (_C18:1_)
5	16.76	6.68	294	C_19_H_34_O_2_	Linoleic acid, methyl ester (C_18:2_)
6	16.87	7.67	296	C_19_H_36_O_2_	11-Octadecenoic acid, methyl ester (C_16:1_)
7	17.42	5.04	298	C_19_H_38_O_2_	Stearic acid methyl ester (C_18:0_)
8	20.77	0.43	292	C_19_H_32_O_2_	Linolenic acid, methyl ester (C_18:3_)
9	21.05	0.55	324	C_21_H_40_O_2_	Gondoic acid, methyl ester (C_20:1_)
10	21.57	10.27	326	C_21_H_42_O_2_	Arachidic acid, methyl ester(C_20:0_)
11	23.55	0.20	340	C_22_H_44_O_2_	Heneicosanoic acid, methyl ester (C_21:0_)
12	25.45	1.43	354	C_23_H_46_O_2_	Behenic acid, methyl ester (C_22:0_)
13	29.06	0.13	382	C_25_H_50_O_2_	Lignoceric Acid methyl ester (C_24:0_)

#### Identification of the phenolic components

**Fig. 3:**
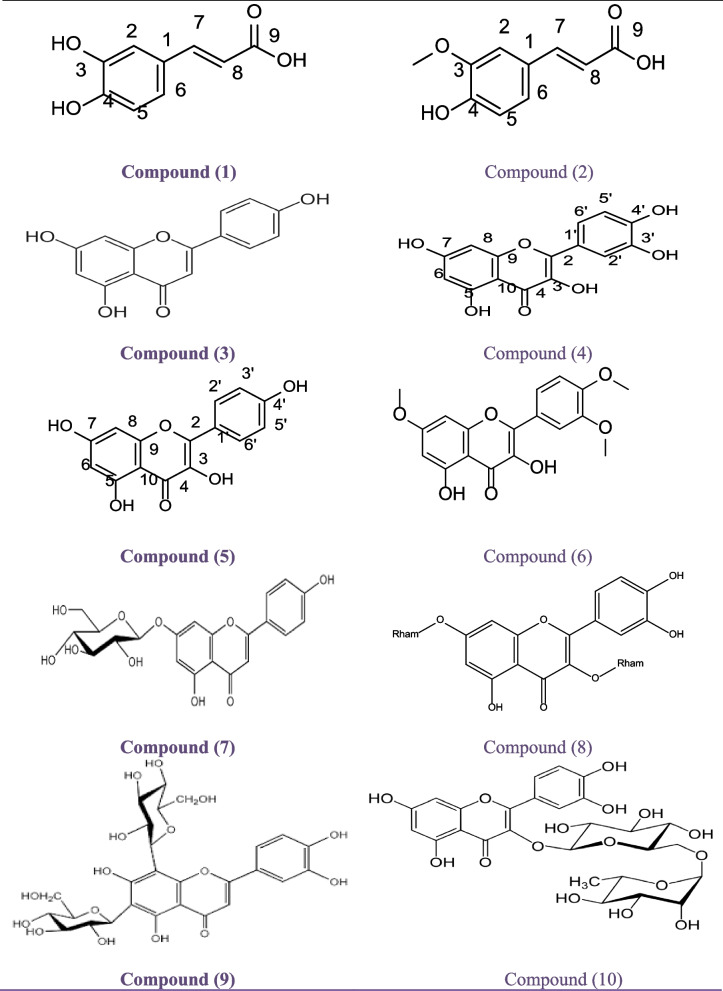
Chemical structures of the phenolic compounds isolated from different extracts of *Casuarina equistefolia* needles

Eight flavonoidal compounds and two phenolic acids were isolated from different column chromatography techniques of chloroform, ethyl acetate, and butanol extracts see Fig. 3. The chloroform extract led to the identification of two phenolic acids and one flavonoidal compound, while the ethyl acetate and butanol extracts resulted in the isolation of four and three flavonoidal compounds, respectively. The structures of these compounds were determined through color reactions, R_*f*_ values, chemical investigations such as acid and enzymatic hydrolysis, and spectral measurements including UV, NMR, and EI/MS. The spectroscopic data were compared with previously published values to confirm their identities [23, 24]. The compounds were identified as caffeic acid (1), ferulic acid (2), apigenin (3), quercetin (4), kaempferol (5), 3,5-dihydroxy-7,3′,4′-trimethoxyflavone (6), apigenin-7-*O*-glucoside (7), kaempferol-3,7-*O*-dirhamnoside (8), luteolin 6,8-di-*C*-glucoside (9), and quercetin-3-*O*-α-rhamnosyl (1′′′→6′′) β-glucoside (10).

A yellowish-brown powder compound C1, was obtained in a quantity of 43 mg, has an R_*f*_ value of 0.70 in BAW and an R_*f*_ value of 0.55 in 15% acetic acid. The UV λmax (nm) of C1 in MeOH is 270 and 268, indicating that it has a phenolic nature when sodium methoxide (NaOMe) is added. The molecular formula of C-1 is C_9_H_8_O_4_, as confirmed by EI/MS with an m/z of 180. The ^1^H-NMR (500 MHz, CD_3_OD) spectrum of C1 shows peaks at δppm 7.65 (1H, d, J = 2.0 Hz, H-2), 6.24 (1H, d, J = 9.2 Hz, H-5), 6.38 (1H, dd, J = 8.2, 2.0 Hz, H-6), 7.21 (1H, d, J = 15.9 Hz, H-7), and 6.12 (1H, d, J = 15.9 Hz, H-8). By comparing this data with literature data [25], C1 was confirmed to be caffeic acid.

A colorless powder compound C2, was obtained in a quantity of 15 mg, with an R_*f*_ value of 0.48 in BAW and an R_*f*_ value of 0.8 in 15% acetic acid. The molecular formula of C2 is C_10_H_10_O_4_, as confirmed by EI/MS with an m/z of 194. The ^1^H-NMR (500 MHz, CD_3_OD) spectrum of C2 shows peaks at δppm 7.67 (1H, d, J = 2.0 Hz, H-2), 9.12 (C-3, OH, s, H-4), 6.42 (1H, d, J = 8.0 Hz, H-5), 6.65 (1H, dd, J = 8.0, 2.0 Hz, H-6), 6.98 (1H, d, J = 16.0 Hz, H-7), 6.12 (1H, d, J = 16.0 Hz, H-8), and 3.60 (3H, s, O-Me). The ^13^C-NMR (125 MHz, CD_3_OD) spectrum of C2 shows peaks at δppm 127.1, 110.8, 148.7, 150.0, and 115.7 for C (1, 2, 3, 4, 5), and 123.5, 146.2, 116.1, and 170.8 for C (6, 7, 8, 9), and 56.2 for O-Me. By comparing this data with literature data [26], C2 was confirmed to be ferulic acid.

A yellow shapeless compound C3, was found and its R_*f*_ values were measured in two solvents, 15% acetic acid and BAW (3:1:1), showing that it is an aglycone. The data gathered from UV, MS, and ^1^H-NMR analyses were identical to the data reported for apigenin in a previous study by [27].

A yellow powder, compound C4 (also known as quercetin) was obtained in a quantity of 25 mg. Its behavior in various solvent systems was analyzed through chromatography on PC, which showed that it is an aglycone (with R_*f*_ values of 0.69 in BAW and 0.21 in 15% acetic acid). The spectra measured for the compounds were consistent with those reported for quercetin in a previous study conducted by [27].

A yellow shapeless substance, C5, was obtained in a quantity of 30 mg. Its behavior in different solvent systems was analyzed through chromatography on PC, which revealed that it is an aglycone (with R_*f*_ values of 0.72 in BAW and 0.11 in 15% acetic acid). The compound's UV spectra were measured in various solvents (MeOH, NaOMe, AlCl_3_, AlCl_3_/HCl, NaOAC, NaOAC/H_3_BO_3_), and its EI/MS and ^1^H-NMR spectra were obtained as well. Based on the obtained data, the compound was identified as kaempferol and the data was consistent with that reported in a previous study by [28].

A yellow powder, compound C6, was obtained in a quantity of 20 mg. Its behavior in different solvent systems was analyzed through chromatography on PC, which revealed that it has R_*f*_ values of 0.85 in BAW and 0.28 in 15% acetic acid. The compound's UV spectra were measured in various solvents (MeOH, NaOMe, AlCl_3_, AlCl_3_/HCl, NaOAC, NaOAC/H_3_BO_3_), and its EI/MS and ^1^H-NMR spectra were obtained as well. Based on the obtained data, the compound was identified as 3,5 dihydroxy- 7,3', 4'-tri methoxy flavone, and the data was consistent with that reported in a previous study by Elgindi et al. in 2016. The compound has a molecular formula of C_18_H_16_O_7,_ and its ^1^H-NMR spectra showed signals at 7.50, 7.22, 7.08, 6.80, and 6.53 ppm, which are attributed to various protons in the molecule. The compound also showed three signals corresponding to three methoxy groups at 3.70, 3.66, and 3.63 ppm [29]. Compound C7 was identified as apigenin-7-*O*-glucoside, a yellow amorphous powder weighing 35 mg. Its chromatographic behavior on PC in different solvent systems indicated that it is glycosidic, with R_*f*_ values of 0.36 in BAW and 0.28 in 15% acetic acid. Complete acid hydrolysis yielded apigenin as an aglycone and glucose as the sugar moiety. UV analysis showed λ_max_ values of 268, 291, and 333 nm in MeOH, and 269, 314, and 382 nm in NaOMe, among others. EI/MS analysis revealed a primary ion with m/z 432 and a secondary ion with m/z 270 for the aglycone apigenin, indicating the formula C_21_H_20_O_10_. ^1^H-NMR analysis showed peaks at δ ppm 7.92 (2H,d, J = 8.2 Hz H-2,6), 6.90 (2H, d, J = 8.15 Hz, H-3', H-5'), 6.82 (1H, d, J=2.2 Hz, H-8), 6.43(1H, d, J= 2.2 Hz, H-6), 5.44 (1H, d, J=7.4 Hz, H-1"), and 3.17-3.47 (5H, m, H-3"–H-6"). The data matched the characteristics reported in the literature [27]. The compound C8, also known as kaempferol-3,7-*O*-dirhamnoside, is a yellow amorphous powder with a weight of 16 mg and a yield of 25 mg. It exhibits glycosidic behavior based on its chromatographic properties in various solvent systems, with an R_*f*_ value of 0.36 in BAW (3:1:1) and 0.70 in 15% acetic acid. Upon complete acid hydrolysis, kaempferol is released as the aglycone, while rhamnose is the sugar moiety. Its UV spectrum shows absorption peaks at 254, 264, and 350 nm in MeOH, 254, 269, and 412 nm in NaOMe, 261, 305, 364, and 400 nm in AlCl_3_, and 253, 270, and 349 nm in NaOAc/H_3_BO_3_, among others. Its EI/MS spectrum exhibits a molecular ion peak at m/z 610 corresponding to the molecular formula C_27_H_30_O_16_, with secondary ions at m/z = 432 and m/z = 286 corresponding to the loss of rhamnose moieties. Its ^1^H-NMR spectrum in CD_3_OD shows signals at δ ppm 7.77, 6.9, 6.7, 6.45, 5.5, 5.32, 1.3, and 0.9, which are consistent with the published data [30]. The compound C9, luteolin 6,8-di-*C*-glucoside, also known as Lucenin-2, is a yellowish powder with a glycosidic nature. Its behavior on paper shows an Rf value of 0.30 in BAW (3:1:1) and an R_*f*_ value of 0.62 in 15% acetic acid. Its UV spectrum in various solvents such as MeOH, NaOMe, AlCl_3_, AlCl_3_/HCl, NaOAC, and NaOAC/H_3_BO_3_ shows absorption maxima at different wavelengths. Its molecular formula is C_27_H_30_O_16_, and its EI/MS spectrum shows a molecular ion peak at m/z 610. Its ^1^H-NMR spectrum in DMSO-d6 shows signals at δ ppm 7.55, 7.48, 6.88, 6.69, 4.76, and 4.81. It is identified as luteolin 6,8-di-*C*-glucoside (Lucenin-2) by responding to enzymatic hydrolysis with *β*-glucosidase and resisting acid hydrolysis, indicating it is in *C*-glycosidic linkage. This identification is consistent with previously reported data [15]. The compound C10, Quercetin-3-*O*-α-rhamnosyl (1'''→6''') β-glucoside, also known as rutin, is a yellow amorphous powder weighing 25 mg. Its chromatographic behavior on PC in different solvent systems shows an R_*f*_ value of 0.38 in BAW (3:1:1) and an R_*f*_ value of 0.66 in 15% acetic acid, indicating its glycosidic nature. Upon complete acid hydrolysis, it yields quercetin as an aglycone, rhamnose, and glucose as sugar moieties. Its UV spectrum in various solvents such as MeOH, NaOMe, AlCl_3_, AlCl_3_/HCl, NaOAc, and NaOAc/H_3_BO_3_ shows absorption maxima at different wavelengths. Its molecular formula is C_27_H_30_O_16_, and its EI/MS spectrum shows a molecular ion peak at m/z 610. Its ^1^H-NMR spectrum in DMSO-d6 shows signals at δ ppm 7.50, 6.80, 6.34, 6.15, 5.11, 4.33, 3.90-3.20, and 0.95. It is identified as quercetin-3-*O*-α-rhamnosyl (1'''→6''') β-glucoside (rutin), which is consistent with previously reported data [31].

#### Quantitative analysis of the phytochemicals

Table 3 presents the findings on the total phenolic and flavonoid contents, as well as antioxidant activity, of various extracts of *C. equistefolia* needles. The methanolic extract (10 CE) had the highest total phenolic content (58.44±0.37 mg/g gallic acid eq.), followed by the ethyl acetate extract (8 CE) (49.33±0.32 mg/g gallic acid eq.) and butanol extract (9 CE) (45.02±0.30 mg/g gallic acid eq.). The total phenolic contents of the chloroform extract (7 CE), mother liquor (11 CE), and hexane extract (12 CE) were (41.21±0.29, 35.71±0.25, and 27.53±0.18 mg/g gallic acid eq.), respectively. Meanwhile, the methanolic extract (10 CE) had the highest total flavonoid content (32.05±0.30 mg/g quercetin eq.), while the hexane extract (12 CE) had the lowest total flavonoid content (15.73±0.22 mg/g quercetin eq.). The results also revealed that antioxidant activity increased as the absorption at 517 nm decreased. The methanolic extract (10 CE) demonstrated the most potent antioxidant activity, accounting for about 86.32%, followed by the ethyl acetate extract (8 CE) at 82.65%.

**Table 3:** TPC, TFC and antioxidant activity of different extracts of *C. equisetifolia*

**Samples**	**(TPC) (mg/g gallic acid eq.)**	**(TFC) (mg/g quercetin eq.)**	**Antioxidant activity (%)**
Chloroform extract (7 CE)	41.21±0.29	18.31±0.25	70.11±0.22
Ethyl acetate extract (8 CE)	49.33±0.32	21.42±0.19	82.65±0.31
Butanol extract (9 CE)	45.02±0.30	20.12±0.28	81.23±0.17
Methanol extract (10 CE)	58.44±0.37	32.05±0.30	86.32±0.17
Mother liquor (11CE)	35.71±0.25	19.54±0.26	58.66±0.25
Hexane extract (12 CE)	27.53±0.18	15.73±0.22	49.22±0.19

#### Antimicrobial activity

**Fig. 4:**
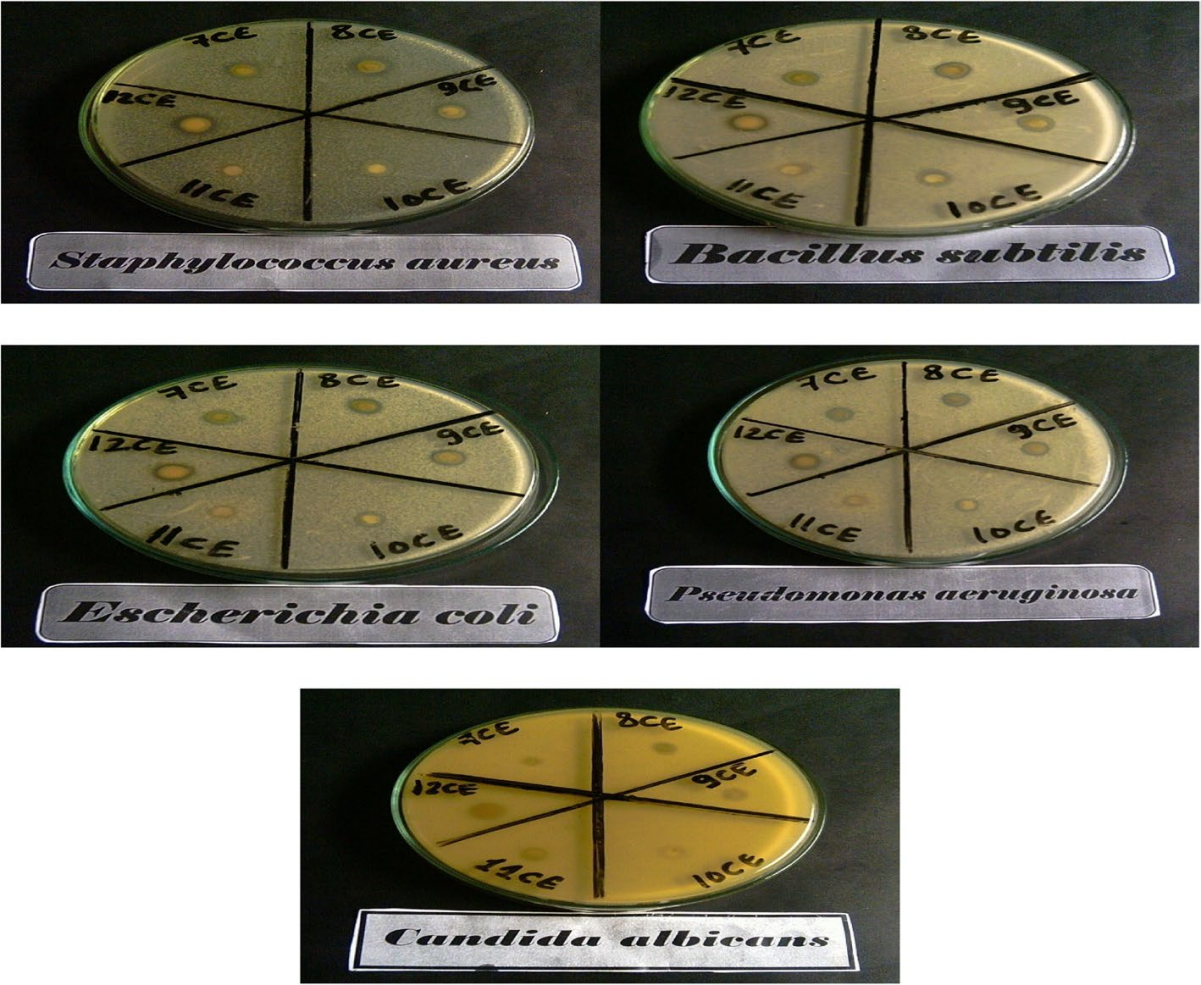
Different *C. equistefolia* extracts were tested using a modified Kirby-Bauer disc diffusion method for their antimicrobial effects against Gram-positive bacteria like *Staphylococcus aureus* (ATCC 12600) and *Bacillus subtilis* (ATCC 6051), as well as Gram-negative bacteria like *Escherichia coli* (ATCC 11775) and *Pseudomonas aeruginosa* (ATCC 10145) and *Candida albicans* (ATCC 7102)

The researchers screened six extracts for their antimicrobial activity against six microorganisms, including two Gram-positive bacteria, two Gram-negative bacteria, and two fungal species, and recorded the size of the inhibition zones in Table 4 and Fig. 4. The ethyl acetate extract (8 CE) demonstrated a larger inhibition zone ranging from 13 to 16 mm against Gram-positive (*Bacillus subtilis* and *Staphylococcus aureu*) respectively, and Gram-negative bacteria (*P. aeruginosa* and *E. coli*), with a clear zone diameter ranging from 12 to 13 mm. respectively. The hexane extract (12 CE) demonstrated effective antimicrobial activity against Gram-positive bacteria with a range of clear zone diameters between 13 to 15 mm, and against Gram-negative bacteria with a range of 12 to 13 mm. Additionally, with a zone of inhibition of 12 mm. ranging from 13 to 14 mm against both Gram-positive and Gram-negative bacteria, the butanol extract (9 CE) also demonstrated antibacterial activity. The chloroform extract (7 CE) also showed antibacterial activity, with clear zone widths of 12 to 13 mm against Gram-negative bacteria and 13 to 14 mm against Gram-positive bacteria (with a zone of inhibition of 12 mm). The study found no inhibitory action against Gram-negative bacteria (*E. coli*), but found modest antibacterial activity against Gram-positive bacteria (*S. aureus*) and Gram-negative bacteria (*P. aeruginosa*) with methanolic extract (10 CE) of *Casuarina equisetifolia* needles. The mother liquor extract (11 CE) showed less inhibitory activity compared to other extracts against both Gram-positive and Gram-negative bacteria. None of the extracts showed inhibitory activity against fungal species *Aspergillus flavus* and *Candida albicans*.

**Table 4:** Antimicrobial activity of the different extracts of *C. equistefolia* needles against *Staphylococcus aureus* (ATCC 12600) and *Bacillus subtilis* (ATCC 6051) were used as Gram-positive bacteria; *Escherichia coli* (ATCC 11775) and *Pseudomonas aeruginosa* (ATCC 10145) were used as Gram-negative; *Candida albicans* (ATCC 7102) and *Aspergillus flavus* (ATCC 9643) were used as fungal species

**Samples**		**Inhibition zone diameter (mm/mg Sample)**					
		**Bacterial species**				**Fungal species**	
		**G**^**+**^		**G ˉ**			
		***B. subtilis***	***S. aureus***	***E. coli***	***P. aeruginosa***	***A. flavus***	***C. albicans***
Standards	Ampicillin	31	24	30	28	-	**-**
	Amphotericin B	-	-	-	-	16	21
Chloroform extract (7 CE)		12	12	12	11	0.0	0.0
Ethyl acetate extract (8 CE)		13	16	13	12	0.0	0.0
Butanol extract (9 CE)		13	14	12	12	0.0	0.0
Methanol extract (10 CE)		12	10	10	10	0.0	0.0
Mother liquor (11CE)		10	10	10	10	0.0	0.0
Hexane extract (12 CE)		13	15	13	12	0.0	0.0

#### Cytotoxic activity

The resulting IC_50_ values, presented in Table 5, reveal that these extracts displayed an inhibitory effects on the viability of the two cell lines at different concentrations. As the IC_**50**_ values decreased, the inhibitory activity of the extracts increased. Notably, the methanol extract (10 CE) exhibited the most potent cytotoxic effect on both cell lines, with the lowest IC_50_ values recorded at 3.68 µg/mL and 5.59 µg/mL for HepG-2 and HCT-116, respectively, that should be recommended for further clinical studies. The chloroform extract (7 CE) displayed high cytotoxic activity on both the HepG-2 and HCT-116 cancer cell lines, with low IC_50_ values of 11.2 µg/mL and 14.6 µg/mL, respectively.

**Table 5:** Cell Viability Inhibition (IC_50_ ± S.D., µg/mL) of different extracts on HepG-2 (liver carcinoma cell line) and HCT-116 (colon carcinoma cell line)

**Samples**	**HepG-2 (IC**_**50**_**) ± S.D. µg/ mL**	**HCT-116 (IC**_**50**_**) ± S.D. µg/ mL**
Chloroform extract (7 CE)	11.2± 0.5	14.6 ± 0.8
Ethyl acetate extract (8 CE)	15.3 ± 1.3	27.5 ± 1.9
Butanol extract (9 CE)	28.9 ± 2.1	44.5 ± 3.7
Methanol extract (10 CE)	3.68 ± 0.1	5.59 ± 0.3
Mother liquor (11 CE)	239 ± 10.3	208 ± 8.6
Hexane extract (12 CE)	204 ± 9.8	153 ± 7.4
Vinblastine Sulfate (Standard)	2.59 ± 0.09	3.15 ± 0.7

The ethyl acetate extract (8 CE) showed moderate cytotoxic activity on both cell lines, with IC_50_ values of 15.3 µg/mL and 27.5 µg/mL for HepG-2 and HCT-116, respectively. The butanol extract (9 CE) exhibited the least cytotoxic activity on both cell lines, with IC_50_ values of 28.9 µg/mL and 44.5 µg/mL for HepG-2 and HCT-116, respectively.

In contrast, the hexane extract (12 CE) displayed minimal cytotoxic activity on both the HepG-2 and HCT-116 cancer cell lines, with IC_50_values of 204 µg/mL and 153 µg/mL, respectively. Similarly, the mother liquor fraction (11 CE) exhibited feeble cytotoxic activity against both cell lines, with IC_50_values of 239 µg/mL and 208 µg/mL for HepG-2 and HCT-116, respectively.

**Fig. 5:**
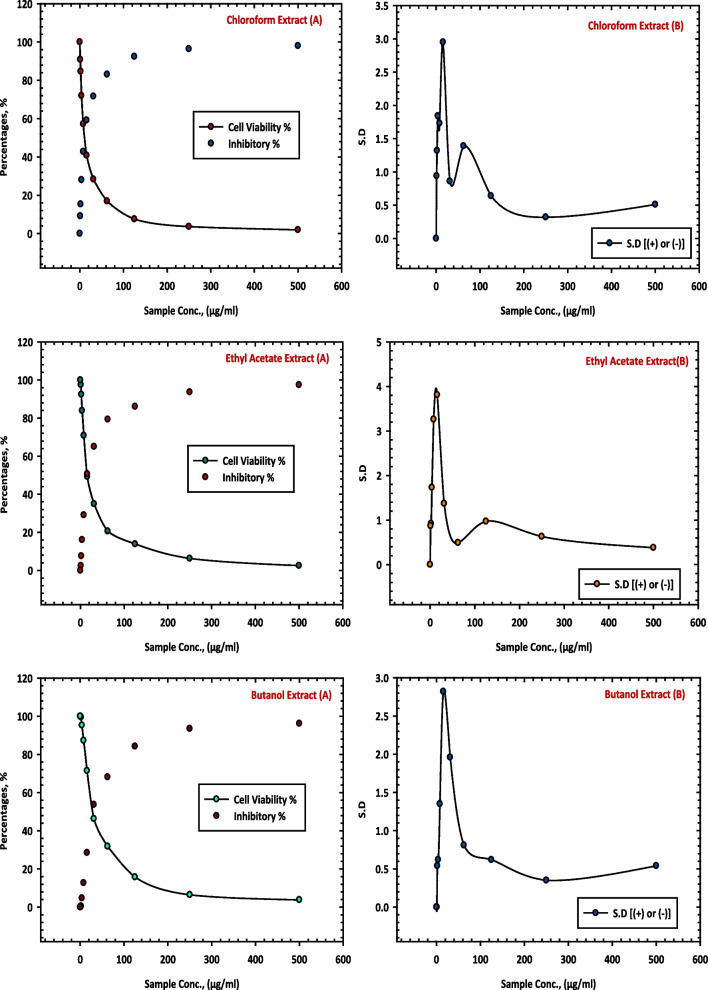
Inhibition of HepG-2 cells by chloroform extract (7 CE), ethyl acetate extract (8 CE) and butanol extract (9 CE) of *C. equistefolia.* **A** represents the relationship between sample concentrations vs the cell viability, and the relationship between sample concentrations vs the inhibitory percentages, and (**B**) represents the relationship between sample concentrations vs the standard deviations

Table 6 presents the results of this study, demonstrating the inhibitory impact of the different extracts against HepG-2 cell line. The chloroform extract (7 CE), ethyl acetate extract (8 CE) and butanol extract (9 CE) from *C. equistefolia* showed 98.03%, 97.46% and 96.18% inhibition of the HepG-2 cells respectively, as shown in Fig. 5, which illustrate the relationship between sample concentrations and cell viability, revealing a gradual reduction in cell viability as the extract concentration increases.

**Table 6:** Inhibition of HepG-2 cells by chloroform extract (7 CE), ethyl acetate extract (8 CE) and butanol extract (9 CE) of *C. equistefolia*

**Sample conc. (µg/mL)**	**Chloroform Extract (7 CE)**			**Ethyl Acetate Extract (8 CE)**			**Butanol Extract (9 CE)**		
	Viability %	Inhibitory %	S.D. (±)	Viability %	Inhibitory %	S.D. (±)	Viability %	Inhibitory %	S.D. (±)
**500**	1.97	98.03	0.51	2.54	97.46	0.38	3.82	96.18	0.54
**250**	3.64	96.36	0.32	6.31	93.69	0.63	6.49	93.51	0.35
**125**	7.58	92.42	0.64	13.89	86.11	0.97	15.76	84.24	0.62
**62.5**	16.92	83.08	1.39	20.63	79.37	0.49	31.85	68.15	0.81
**31.25**	28.36	71.64	0.86	34.95	65.05	1.37	46.32	53.68	1.96
**15.6**	40.83	59.17	2.95	49.27	50.73	3.81	71.46	28.54	2.82
**7.8**	57.21	42.79	1.73	70.88	29.12	3.26	87.29	12.71	1.35
**3.9**	71.98	28.02	1.84	83.91	16.09	1.73	95.30	4.7	0.62
**2**	84.65	15.35	1.32	92.44	7.56	0.92	99.48	0.52	0.54
**1**	90.84	9.16	0.94	97.52	2.48	0.87	100	0	0
**0**	100	0	0	100	0	0	100	0	0

**Fig. 6:**
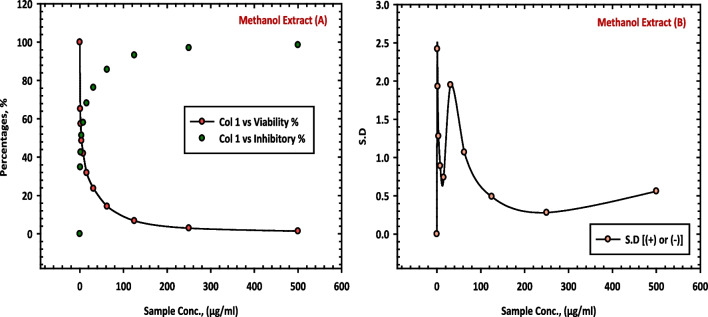
Inhibition of HepG-2 cells by methanol extract (10 CE) of *C. equistefolia.* **A** represents the relationship between sample concentrations vs the cell viability, and the relationship between sample concentrations vs the inhibitory percentages, and (**B**) represents the relationship between sample concentrations vs the standard deviations

**Fig. 7:**
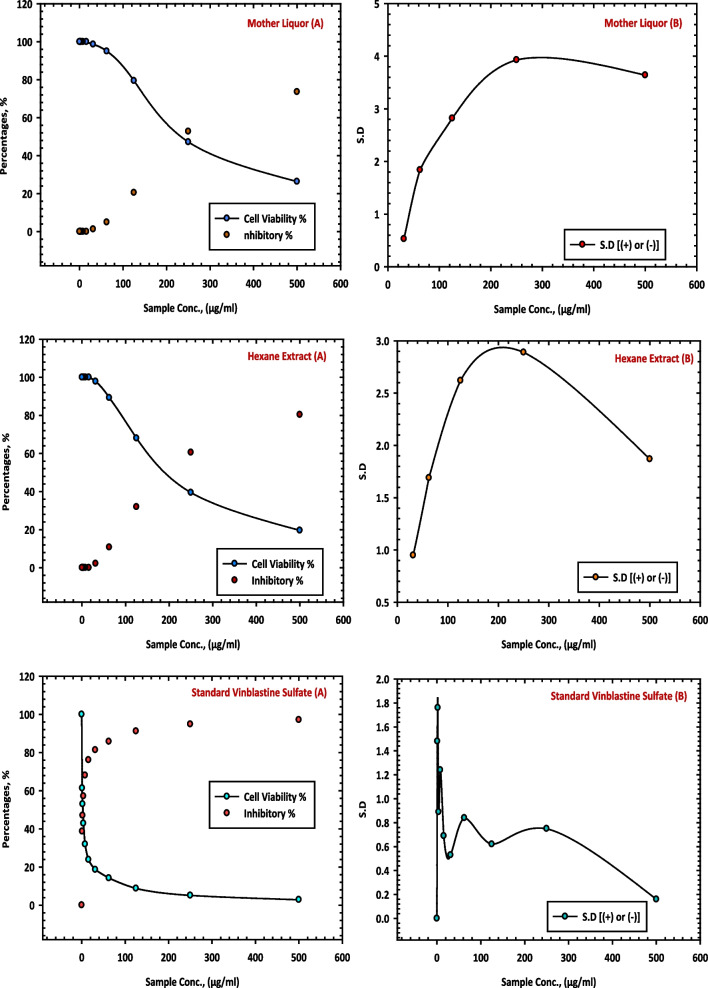
Inhibition of HepG-2 cells by mother liquor (11 CE), hexane extract (12 CE) and standard vinblastine sulfate of *C. equistefolia.* **A** represents the relationship between sample concentrations vs the cell viability, and the relationship between sample concentrations vs the inhibitory percentages, and (**B**) represents the relationship between sample concentrations vs the standard deviations

The results of this study, as presented in Table 7, provide evidence of the inhibitory effects of the methanol extract (10 CE), mother liquor (11 CE) and hexane extract (12 CE) of *C. equistefolia* on HepG-2 cells in comparable with the standard vinblastine sulfate. The methanol extract (10 CE), from *C. equistefolia* showed 98.57% inhibition of the HepG-2 cells as shown in Fig. 6. The mother liquor (11 CE) and hexane extract (12 CE) of *C. equistefolia* on HepG-2 cells displayed 73.62% and 80.38% inhibition of the HepG-2 cells respectively, as shown in Fig. 7.

**Table 7:** Inhibition of HepG-2 cells by methanol extract (10 CE), mother liquor (11 CE), hexane extract (12 CE) of *C. equistefolia* and standard vinblastine sulfate

**Sample conc. (µg/ mL)**	**10 CE**		**11 CE**		**12 CE**		**Standard Vinblastine Sulfate**	
	**Viab. %**	**Inhib. % (±) S.D.**	**Viab. %**	**Inhib. % (±) S.D.**	**Viab. %**	**Inhib. % (±) S.D.**	**Viab. %**	**Inhib. % (±) S.D.**
500	1.43	98.57±0.56	26.38	73.62±3.64	19.62	80.38±1.87	2.86	97.14±0.16
250	2.96	97.04±0.28	47.21	52.79±3.93	39.47	60.53±2.89	5.13	94.87±0.75
125	6.78	93.22±0.49	79.46	20.54±2.82	68.04	31.96±2.62	8.79	91.21±0.62
62.5	14.29	85.71±1.07	95.02	4.98±1.84	89.23	10.77±1.69	14.20	85.8±0.84
31.25	23.67	76.33±1.95	98.69	1.31±0.53	97.81	2.19±0.95	18.65	81.35±0.53
15.6	31.82	68.18±0.74	100	0	100	0	23.87	76.13±0.69
7.8	41.95	58.05±0.89	100	0	100	0	31.94	68.06±1.24
3.9	48.60	51.4±1.28	100	0	100	0	42.85	57.15±0.89
2	57.41	42.59±1.93	100	0	100	0	52.98	47.02±1.76
1	65.20	34.8±2.42	100	0	100	0	61.32	38.68
0	100	0	100	0	100	0	100	0

The findings suggest that the feeble potential inhibitory effects of mother liqour (11 CE) and hexane extract (12 CE) of *C. equistefolia* on HepG-2 cells (Table 7, Fig. 7), in comparable with the standard vinblastine sulfate may hold promise as a potential inhibitor of HepG-2 cell proliferation, with increasing concentrations of the extract. Nonetheless, vinblastine sulfate is a well-known chemotherapeutic drug with documented efficacy against various cancer types, including liver cancer (Table 7, Fig. 7).

**Fig. 8:**
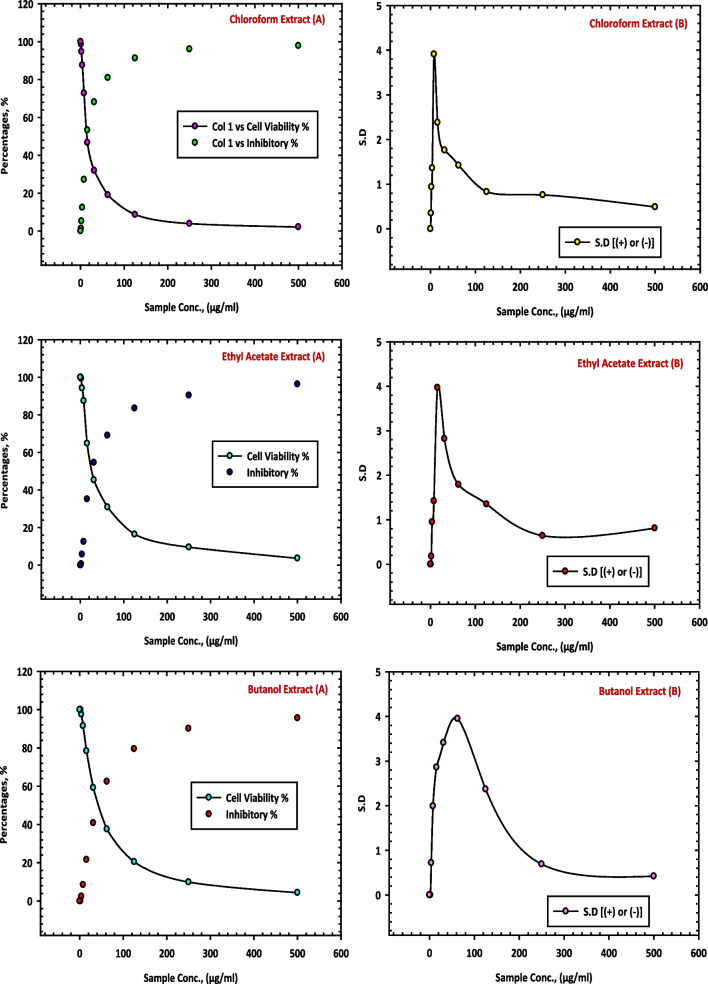
Inhibition of HCT-116 ells by chloroform extract (7 CE), ethyl acetate extract (8 CE) and butanol extract (9 CE) of *C. equistefolia.* **A** represents the relationship between sample concentrations vs the cell viability, and the relationship between sample concentrations vs the inhibitory percentages, and (**B**) represents the relationship between sample concentrations vs the standard deviations

The results of this study demonstrated the inhibitory effects of the chloroform extract (7 CE), ethyl acetate extract (8 CE) and butanol extract (9 CE) of *C. equistefolia* on HCT-116 cells (Table 8). The chloroform extract (7 CE), ethyl acetate extract (8 CE) and butanol extract (9 CE) from *C. equistefolia* showed 97.85%, 96.33% and 95.64% inhibition of the HCT-116 cells respectively, as shown in Fig. 8, which illustrate the relationship between sample concentrations and cell viability, revealing a gradual reduction in cell viability as the extract concentration increases.

**Table 8:** Inhibition of of HCT-116 cells by chloroform extract (7 CE), ethyl acetate extract (8 CE) and butanol extract (9 CE) of *C. equistefolia*

**Sample conc. (µg/mL)**	**Chloroform Extract (7 CE)**			**Ethyl Acetate Extract (8 CE)**			**Butanol Extract (9 CE)**		
	**Viability %**	**Inhibitory %**	**S.D. (±)**	**Viability %**	**Inhibitory %**	**S.D. (±)**	**Viability %**	**Inhibitory %**	**S.D. (±)**
500	2.15	97.85	0.49	3.67	96.33	0.81	4.36	95.64	0.42
250	3.92	96.08	0.76	9.58	90.42	0.64	9.87	90.13	0.69
125	8.71	91.29	0.83	16.47	83.53	1.35	20.49	79.51	2.37
62.5	19.06	80.94	1.42	30.92	69.08	1.79	37.56	62.44	3.95
31.25	31.92	68.08	1.76	45.36	54.64	2.82	59.21	40.79	3.41
15.6	46.70	53.3	2.38	64.81	35.19	3.97	78.42	21.58	2.86
7.8	72.83	27.17	3.91	87.50	12.5	1.42	91.53	8.47	1.99
3.9	87.56	12.44	1.36	94.23	5.77	0.95	97.60	2.4	0.72
2	94.72	5.28	0.94	99.34	0.66	0.18	100	0	0
1	98.67	1.33	0.35	100	0	0	100	0	0
0	100	0	0	100	0	0	100	0	0

**Fig. 9:**
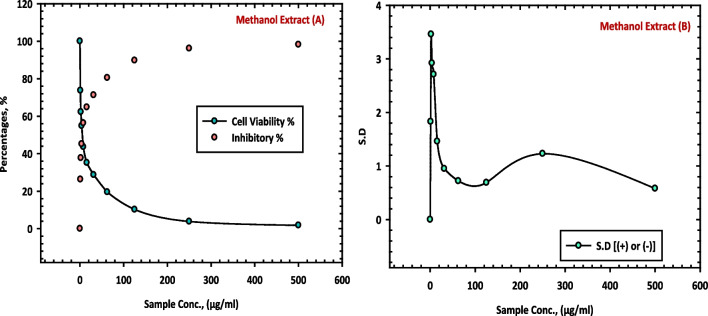
Inhibition of HCT-116 cells by methanol extract (10 CE) of *C. equistefolia.* **A** represents the relationship between sample concentrations vs the cell viability, and the relationship between sample concentrations vs the inhibitory percentages, and (**B**) represents the relationship between sample concentrations vs the standard deviations

**Fig. 10:**
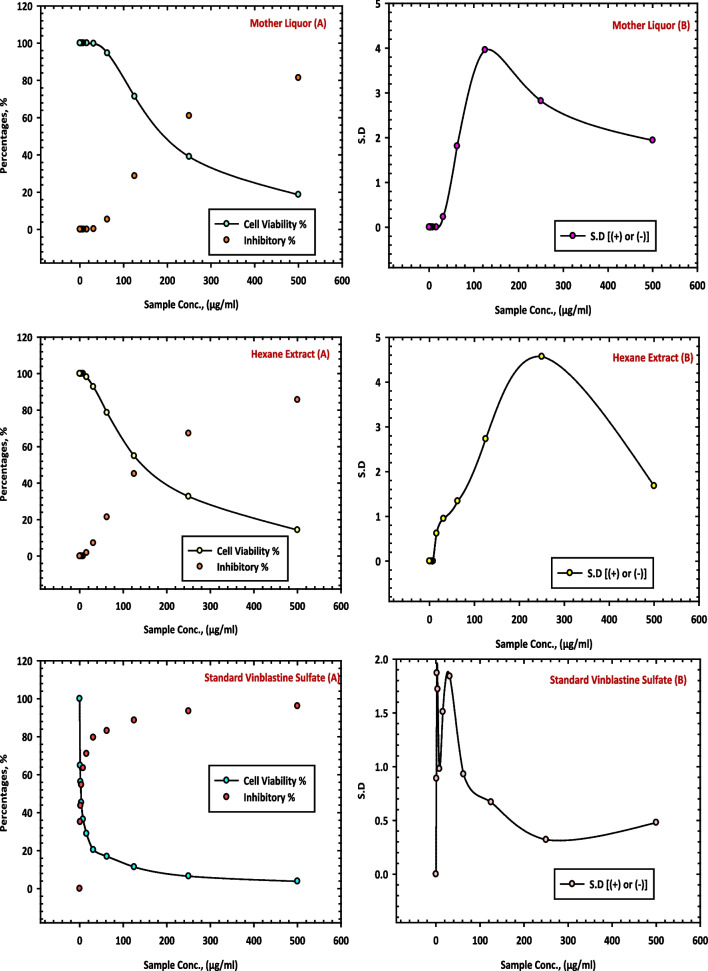
Inhibition of HCT-116 cells by mother liquor (11 CE), hexane extract (12 CE) and standard vinblastine sulfate of *C. equistefolia.* **A** represents the relationship between sample concentrations vs the cell viability, and the relationship between sample concentrations vs the inhibitory percentages, and (**B**) represents the relationship between sample concentrations vs the standard deviations

The results of this study, as presented in Table 9, provide evidence of the inhibitory effects of the methanol extract (10 CE), mother liquor (11 CE) and hexane extract (12 CE) of *C. equistefolia* on HCT-116 cells. The methanol extract (10 CE), from *C. equistefolia* showed 98.21% inhibition of the HepG-2 cells as shown in Fig. 9. The mother liquor (11 CE) and hexane extract (12 CE) of *C. equistefolia* on HCT-116 cells displayed 81.33% and 85.66% inhibition of the HCT-116 cells respectively, as shown in Fig. 10.

**Table 9:** Inhibition of HCT-116 cells by methanol extract (10 CE), mother liquor (11 CE), and hexane extract (12 CE) of *C. equistefolia* and standard vinblastine sulfate

**Sample conc. (µg/ mL)**	**10 CE**		**11 CE**		**12 CE**		**Standard Vinblastine Sulfate**	
	**Viab. %**	**Inhib. % (±) S.D.**	**Viab. %**	**Inhib. % (±) S.D.**	**Viab. %**	**Inhib. % (±) S.D.**	**Viab. %**	**Inhib. % (±) S.D.**
500	1.79	98.21±0.58	18.67	81.33±1.94	14.34	85.66±1.68	3.86	96.14±0.48
250	3.84	96.16±1.23	39.04	60.96±2.82	32.71	67.29±4.57	6.54	93.46±0.32
125	10.23	9.77±0.69	71.32	28.68±3.96	54.89	45.11±2.73	11.38	88.62±0.67
62.5	19.51	80.49±0.72	94.63	5.37±1.81	78.62	21.38±1.34	16.89	83.11±0.93
31.25	28.74	71.26±0.95	99.75	0.25±0.23	92.79	7.21±0.95	20.42	79.58±1.84
15.6	35.20	64.8±1.46	100	0	98.14	1.86±0.62	28.97	71.03±1.51
7.8	43.65	56.35±2.71	100	0	100	0	36.48	63.52±0.98
3.9	54.86	45.14±2.92	100	0	100	0	45.36	54.64±1.72
2	62.30	37.7±3.46	100	0	100	0	56.31	43.69±1.87
1	73.69	26.31±1.83	100	0	100	0	64.87	35.13±0.89
0	100	0	100	0	100	0	100	0

The findings suggest that the feeble potential inhibitory effects of mother liqour (11 CE) and hexane extract (12 CE) of *C. equistefolia* on HCT-116 cells (Table 9, Fig. 10), in comparable with the standard vinblastine sulfate.

## Discussion

One of the ways to evaluate the potential therapeutic properties of *Casuarina equistefolia* is by measuring its total phenolic and flavonoid contents. Phenolic compounds are known to possess antioxidant properties, and they have been shown to play a role in preventing oxidative stress-related diseases, such as cancer, cardiovascular disease, and neurodegenerative disorders. Overall, evaluating the TPC, phytochemical profile, and potential therapeutic properties of different extracts of *Casuarina equistefolia* can provide valuable insights into the plant's potential as a source of novel therapeutics. The variation in the quantitative analysis of the different extracts may be attributed to the solvent polarity and the ratio of solute to the solvent used in the extraction process [32]. In addition to their relative amounts and composition, phenolics and flavonoids' antioxidant potency is also influenced by their concentration, degree of polymerization, and interactions with various colorimetric tests [33]. Antioxidants are compounds that can prevent or delay oxidative damage caused by free radicals in the body. *Casuarina equisetifolia* has been found to possess significant antioxidant activity due to its high content of phenolic compounds and flavonoids. The ethyl acetate and methanol extracts exhibited significant antioxidant activity, attributed to the presence of phenolic and flavonoid compounds [34].

*Casuarina equisetifolia* extracts have been reported to exhibit antimicrobial activity against a wide range of pathogenic bacteria and fungi. Antimicrobial activity is attributed to the presence of various secondary metabolites such as flavonoids, tannins, and alkaloids [9, 11].

Saranya and Gowrie [34], stated that the antimicrobial properties of the methanol extract of *Casuarina equisetifolia* against these microorganisms may be attributed to the presence of phenolic compounds, alkaloids, saponin, and flavonoids in the leaf extract, which interacts with extracellular proteins and the bacterial cell wall [35, 36].

Numerous microbial pathogens, cell wall degradation, damage to membrane proteins and cytoplasmic membrane, contents leakage out of the cell, cytoplasm coagulation, and proton motive force depletion are targets of the identified phytochemicals in hexane extract, and these mechanisms of action have been reported [37].

None of the extracts showed inhibitory activity against fungal species *Aspergillus flavus* and *Candida albicans*. These findings align with a previous study by [38], who found that ethanolic extracts had the most significant antifungal activity, with *Aspergillus flavus* being the most susceptible fungus and *Candida albicans* being the most resistant. The study suggests that the presence of phytochemicals, particularly phenolic compounds, may contribute to the potent antimicrobial activity observed in plant extracts [39].

Quorum sensing, inhibition of bacterial virulence, which hinders their ability to form biofilms, inhibition of efflux pumps, inhibition of NADH-cytochrome C reductase activity, and inhibition of ATP synthase are all part of the antimicrobial mechanism of flavonoids like quercetin, naringenin, myricetin, and kaempferol [37]. *Casuarina equisetifolia* extracts have been reported to exhibit antimicrobial activity against a wide range of pathogenic bacteria and fungi.

Natural polyphenolic chemicals are prized for their ability to prevent cancer [40, 41]. All the detected compounds in the non-polar fraction as well as the separated flavonoids and/or phenolic acids from the plant leaves were reported for their cytotoxic and antioxidant properties. MCF-7 cancer cells did not exhibit any growth inhibition in response to polar solvent extracts. However, non-polar solvent extracts were found to induce cell death through apoptosis, which involves DNA fragmentation and the release of caspase 3 [42].

The presence of polyphenols and certain flavonoids, which were identified during the phytochemical screening and have been shown to have substantial antioxidant and anticancer effects, may be the cause of the alcoholic extract's remarkable activity [43].

The results of this study demonstrate the inhibitory effects of the chloroform extract (7 CE), ethyl acetate extract (8 CE) and butanol extract (9 CE) of *C. equistefolia* on HCT-116 cells. It reveals a concentration-dependent decrease in cell viability with increasing concentrations of the chloroform extract, indicating a stronger inhibition of HCT-116 cell growth at higher concentrations (Table 8). This indicates that the chloroform extract contains bioactive components with significant anti-proliferative properties against HCT-116 cells. Overall, these findings suggest that the chloroform extract (7 CE) of *C. equistefolia* holds promise as a potential anti-cancer agent against HCT-116 cells (Fig. 8).

The results of this study, displayed that higher concentrations of the chloroform extract (7 CE), ethyl acetate extract (8 CE) and butanol extract (9 CE) led to a stronger inhibition of HepG-2 and HCT-116 cell growth. Moreover, it also demonstrates that the inhibitory effect of these extracts increase with higher concentrations, suggesting that the active compounds within the chloroform, ethyl acetate and butanol extracts possess significant anti-proliferative properties against HepG-2 and HCT-116 cells. Additionally, it represents the relationship between sample concentrations and standard deviations and provides insight into the consistency of the inhibitory effects across different concentrations (Fig. 8). A lower standard deviation suggests a more consistent inhibitory effect, indicating that the extract's efficacy remains relatively stable throughout the tested concentration range.

In the current investigation, chloroform extract (7 CE) was shown to include phenolic components, including flavonoids (apigenin) and phenolic acids (caffeic and ferulic acids). Numerous of these phenolic compounds have been shown to have chemopreventive and chemotherapeutic effects against liver and colon cancer, and the underlying processes are well understood [44].

The various extracts of *Casuarina equisetifolia's* antioxidant, antibacterial, and cytotoxic properties have molecular explanations based on typical bioactive chemicals present in plants and their recognized effects on microbes and cancer cells.

In numerous *in vitro* and *in vivo* investigations, phenolic acids, a subclass of plant phenolics further separated into benzoic and cinnamic acids, have been linked to powerful anticancer properties. Additionally, the therapeutic effects of phenolic acids are strengthened by their function as epigenetic regulators and promoters of unfavorable side effects or resistance linked to traditional anticancer therapy [45].

Numerous biological activities, including antioxidant and anti-cancer properties, are held by caffeine in nature and its derivatives (caffeic acid phenethylester) [46].

Numerous tumors, including colorectal cancer, breast cancer, liver cancer, lung cancer, melanoma, prostate cancer, and osteosarcoma, have shown apigenin to have wide anticancer effects [47].

Through causing cell death, promoting autophagy, and modifying the cell cycle, this flavone prevents the development of cancer cells. Additionally, apigenin reduces the motility of cancer cells and prevents their migration and invasion. Apigenin has recently been claimed to exhibit anti-cancer properties by triggering an immune response [47].

Apigenin can also increase chemotherapy-induced cell death via regulating the degree of mitochondrial protein expression. Apigenin increased the expression of Bim and decreased the expression of Mcl-1 in the colorectal cancer cell lines HCT116, which combined with the Bcl-2 inhibitor ABT-263 to cause mitochondria-dependent cell death [48].

In the current investigation, ethyl acetate extract (8 CE), was shown to include phenolic components, including flavonoids (quercetin, kaempferol, 3,5-dihydroxy-7,3′,4′-trimethoxyflavone and apigenin-7-*O*-glucoside).

Important flavonoids and phenolics including kaempherol and quercetin, which are recognized as potent antioxidants and have a range of anti-cancer activities, are also present in the ethyl acetate extract [49].

Adults are most frequently diagnosed with hepatocellular carcinoma (HCC), which is a primary liver cancer. Human liver cancer cells (HepG2, SK-HEP-1, Huh7) were shown to be greatly inhibited in their ability to proliferate by kaempferol, and this inhibition was dose-dependent. Kaempferol inhibits cell invasion and migration by inducing cell death and cell cycle arrest in the G2/M phase. By causing the loss of mitochondrial membrane potential, swelling of the mitochondria, and an increase in the quantity of cleaved caspase-3, kaempferol is also capable of releasing cytochrome c [50].

More than 1.8 million new instances of colorectal cancer are reported each year, making it one of the most prevalent diseases in the world. According to reports, kaempferol has cytotoxic effects on a variety of human colorectal cancer cell lines, including LS174-R colon, HCT116, HT-29, HCT-15, and SW480 cells [50].

Due to ROS level modulation-induced apoptosis and S phase arrest-induced flavonol kaempferol reduced the development of malignant bladder cells. In colorectal cancer HCT116, HCT15, and SW480 cell lines, it induced apoptosis by activating caspases as a result of ROS production [51]. Additionally, by targeting mitochondria through ROS, kaempferol has lethal effects on rat hepatocellular carcinoma cells [52].

*In vitro* and *in vivo* models of various malignancies have demonstrated quercetin's ability to exhibit anti-tumor activities through a number of pathways, and the results are encouraging. *In vitro*, quercetin greatly slows down the cell cycle, encourages apoptosis, and blocks angiogenesis and metastasis. The results of *in vivo* investigations suggest that the chosen dosage of quercetin is beneficial in preventing the development of xenograft tumor models. Quercetin-derived bioactive substances have been demonstrated to stop the growth of liver cancer cells [44].

In the current investigation, butanol extract (9 CE), was shown to include phenolic components, including flavonoids (kaempferol-3,7-*O*-dirhamnoside, luteolin 6,8-di-*C*-glucoside, and quercetin-3-*O*-α-rhamnosyl (1′′′→6′′) β-glucoside).

In a separate study, rutin was administered to the SW480 tumor cell line (a human colon cancer cell line), and it was found to enhance the mean survival time by 50 days while also having less detrimental effects on the body and relative organ weight of mice [53].

One of the primary naturally occurring forms of quercetin is quercetin glycosides, which makes them intriguing substances for the treatment of cancer. As a result, several researchers investigated the effects of quercetin derivatives in various cancers, including hepatocellular carcinoma HCC [54].

Similar to quercetin, quercetin glycosides have a pro-apoptotic effect. The apoptosis of cancer cells was eventually brought on by quercetin glycosides, which also increased the production of cytochrome c and controlled the expression of apoptosis-related proteins. In HepG2 cells, quercetin glycosides can also promote caspase-3-induced apoptosis [55].

Antunes-Ricardo et al., demonstrated that isorhamnetin diglycosides were more cytotoxic than pure isorhamnetin aglycone against colon cancer HT-29 cells and that glycosylation impacts the anti-proliferative action of the extract of *Opuntia ficus*-*indica* (L.) Mill. var Jalpa (Cactaceae) [56].

Overall, these findings underscore the potential of the chloroform extract (7 CE), ethyl acetate extract (8 CE) and butanol extract (9 CE) from *C. equistefolia* as a promising candidates for further exploration and development as an anti-cancer agent against HepG-2 and HCT-116 cells.

The methanol extract (10 CE), from *C. equistefolia* showed the most potent inhibition of the HepG-2 and HCT-116 cells (Figs. 6 and 9), respectively, illustrating a dose-dependent decrease in cell viability with increasing concentrations of the extract, indicating that the methanol extract contains bioactive components capable of inhibiting HepG-2 and HCT-116 cell growth.

The fact that herbal medicine formulations frequently contain a number of different ingredients, each of which functions in concert to produce a drug with the best possible biological and clinical effects, is one of its fundamental tenets. In keeping with this, it is uncommon for a single ingredient from herbs or spices with the best biological and clinical action to be separated. As a result, several studies have demonstrated that combining various bioactive components inhibits cancer development synergistically [57].

The findings in our work showed that the examined methanol extracts' combination of phenolic acids and flavonoids, in particular, had a synergistic impact among various bioactive substances that resulted in a potent lethal effect on liver and colon cancer cell lines.

Furthermore, it is also demonstrated that the inhibitory effect becomes more pronounced at higher concentrations, suggesting potent anti-proliferative properties of the methanol extract against HepG-2 and HCT-116 cells. The relationship between sample concentrations and standard deviations, depicted in it, indicates a consistent inhibitory effect of the extract across different concentrations. These findings highlight the potential of the methanol extract (10 CE) of *C. equistefolia* as a promising candidate for further investigation as an anti-cancer agent against HepG-2 and HCT-116 cells.

Similarly, the results of this study, Figs. 7 and 10 displayed weak potential inhibitory effects of the mother liquor (11 CE) of *C. equistefolia* on HepG-2 cells and HCT-116 cells, respectively.

Additionally, the findings of this study suggest feeble potential inhibitory effects of the hexane extract (12 CE) of *C. equistefolia* on HepG-2 cells (Table 7, Fig. 7) and on HCT-116 cells (Table 9, Fig. 10), in comparable with the standard vinblastine sulfate. Nonetheless, Vinblastine sulfate is a well-known chemotherapeutic drug with documented efficacy against various cancer types, including liver cancer.

However, further experimental research is necessary to validate and confirm these findings, including the identification of more specific bioactive compounds and elucidation of the underlying mechanisms of action.

## Conclusion

The effectiveness of predicting the constituents of medicinal plants depends largely on the solvent used during extraction. A recent study on *C. equisetifolia* needles found that different extracts had varying levels of antioxidant activity. The study measured the total phenolic and flavonoid content and discovered that the extracts had different degrees of antimicrobial activity against all tested isolates. Five organic solvents and a mother liquor fraction were used to determine the antimicrobial activity of the *C. equisetifolia* extracts. The ethyl acetate extract (8 CE) was found to have a larger inhibition zone, while the methanol extract (10 CE) showed the most cytotoxic activity against both (HepG-2) and (HCT-116) cancer cell lines with low IC_50_ values (3.68 µg/mL and 5.59 µg/mL, respectively). These findings suggest that the combination of antioxidant antimicrobial and cytotoxic properties make *C. equisetifolia* extracts useful for treating diseases such as cancer and AIDS.

## Data Availability

All data generated or analysed during this study are included in this published article.
